# Effects of Core Self-Evaluations on the Job Burnout of Nurses: The Mediator of Organizational Commitment

**DOI:** 10.1371/journal.pone.0095975

**Published:** 2014-04-22

**Authors:** Yangen Zhou, Jiamei Lu, Xianmin Liu, Pengcheng Zhang, Wuying Chen

**Affiliations:** 1 Education Science College, Shanghai Normal University, Shanghai, People’s Republic of China; 2 Education Department, Nanjing Normal University Taizhou College, Taizhou, People’s Republic of China; University of Leicester, United Kingdom

## Abstract

**Objective:**

To explore the impact of Core self-evaluations on job burnout of nurses, and especially to test and verify the mediator role of organizational commitment between the two variables.

**Method:**

Random cluster sampling was used to pick up participants sample, which consisted of 445 nurses of a hospital in Shanghai. Core self-evaluations questionnaire, job burnout scale and organizational commitment scale were administrated to the study participants.

**Results:**

There are significant relationships between Core self-evaluations and dimensions of job burnout and organizational commitment. There is a significant mediation effect of organizational commitment between Core self-evaluations and job burnout.

**Conclusions:**

To enhance nurses’ Core self-evaluations can reduce the incidence of job burnout.

## Introduction

Job burnout is a state of mental and physical exhaustion caused by work-related stress [1], [2]. Maslach and Jackson defined it as long-term stress response of an individual to prolonged exposure to emotional and interpersonal stressors at work, which encompass emotional exhaustion, depersonalization, and reduced personal accomplishment [3]. Research shows an increasing number of employees experiencing job burnout. In 1997, using Packer’s core evaluation theory as reference, American scholar Judge, et al extracted four core traits with relatively large correlations: self-esteem, generalized self-efficacy, neuroticism, and locus of control [4]. Judge argued that among these four core traits, a higher-order factor, which he called core self-evaluation, exists, and thus he proposed the core self-evaluation theory [4], [5]. According to Judge, core self-evaluation is “an individual’s most fundamental evaluation of his own ability and value.” [6], [7] Core self-evaluation is a lasting evaluation of an individual’s subconscious and a wider and higher-level concept of personality [8]. However, studies on the correlated variable between core self-evaluation and profession are few. According to Judge et al.’s research in 1999, core self-evaluation has a positive correlation with salary, vocational commitment, and the ability to cope with organizational changes [9]. Organizational commitment refers to the attitude towards the organization, which can also be defined as the desires to remain in the organization [4]. A large body of research supports the relationship between organizational commitment and job burnout [10]. For example, Leiter and Maslach found that burnout and organizational commitment are correlated, and both of them can be affected by interpersonal environment [11]. In addition, significant correlations were also found between Core self-evaluations and organizational commitment [4], [9].

Nursing is a typical vocation of providing care and treating illnesses of individuals. Therefore, nurses commonly experience job burnout because of the heavy mental and work load [12]. Research shows that 40% of nurses are unsatisfied with their jobs, and 33% of nurses have varying work goals [13]. Using nurses as the research object, this study investigates the current status of nurses’ core self-evaluation, organizational commitment, and job burnout, probes into the relationship of these three variables, and analyzes how an individual’s core self-evaluation influences job burnout.

## Method

### 2.1 Participants and Procedure

The participants comprised 445 female nurses from four large general hospitals in Beijing, China. Their ages ranged from 20 years to 41 years, with a mean of 24.78 years (SD = 4.10). At the time of the gathering of data, the nurses had worked in hospitals from 6 to 220 months. The participants completed the questionnaire in a classroom environment. All participants signed informed consent forms before completing the measures. The research described in this paper meets the ethical guidelines of the Shanghai Normal University and was approved by the ethics committee of Shanghai Normal University. Participants were told they were engaging in a psychological investigation in which there were no correct or incorrect answers. We distributed 445 questionnaires, which were all collected and validated. Participants received ¥40 in compensation.

### 2.2 Instruments

#### 2.2.1 Core Self-Evaluation Scale (CSES)

The core self-evaluations scale (CSES), which was developed by Judge et al., is a 12-item self-report measure of core self-evaluations [5], [8]. Items are rated from 1 (strongly disagree) to 5 (strongly agree). Examples of items include “I am confident I get the success I deserve in life,” and “Sometimes when I fail, I feel worthless.” The scale scores are the sum of the ratings of the items. Relevant items were reverse-coded. In this study, the Cronbach alpha coefficient for the CSES was 0.742.

#### 2.2.2 Maslach Burnout Inventory-General Survey

The Maslach Burnout Inventory-General Survey (MBI-GS), developed by Maslach, is a 15-item self-report measure of job burnout that includes three dimensions, namely, emotional exhaustion, depersonalization, and reduced personal accomplishment [14]. The items are rated from 1 (never) to 7 (every day). Some items are “I have become less enthusiastic about my work,” and “I have become more cynical about whether my work contributes anything.” In this study, the Cronbach alpha coefficients were 0.804, 0.845, and 0.734, for the three sub-scales in our study.

#### 2.2.3 Organizational Commitment Scale

The Organizational Commitment Scale (OCS), developed by Allen and Meyer, comprises 18 items and three dimensions, namely, affective, normative, and continuance [15]. The items are rated from 1 (strongly disagree) to 6 (strongly agree). Some items are “I am very happy being a member of this organization,” “I worry about the loss of investments I have made in this organization,” and “I feel that I owe this organization quite a bit because of what it has done for me.” Scale scores are the sum of items with reverse coding of relevant items. In this study, the Cronbach alpha coefficients were 0.779, 0.825, and 0.794, for the three sub-scales in our study. [16].

### 2.3 Data Analyses

Data analysis was performed using the SPSS statistical software package. Correlation analysis, regression analysis and path analysis were used. A p-value <0.05 was considered statistically significant. Structural Equation Modeling analysis was performed by AMOS 17.0 program.

## Results

Results of the correlation between each dimensional variable of core self-evaluation and each dimensional variable of organizational commitment are shown in table 1. Results show a significant positive correlation between each dimension of core self-evaluation and each dimension of organizational commitment. A significant positive correlation exists between the total points of core self-evaluation and the point of organizational commitment (p<0.001).

**Table 1:** Correlation analysis between CSE and organizational commitment (r).

	Affect Commitment	Normative Commitment	Cost Commitment	Total Scores
Core self-evaluations	0.356***	0.299***	0.161**	0.334***

Note: **p<0.01; ***p<0.001.

Results of the correlation between each dimensional variable of organizational commitment and each dimensional variable of job burnout are shown in table 2. The results revealed that significant negative correlations are found between other dimensions of organizational commitment and other dimensions of job burnout (p<0.01).

**Table 2:** Correlation analysis between organizational commitment and job burnout (r).

	Emotional Exhaustion	Depersonalization	Reduced Personal Accomplishment	Total Scores
Affect Commitment	−0.594***	−0.546***	−0.544***	−0.600***
Normative Commitment	−0.458***	−0.415***	−0.396***	−0.451***
Cost Commitment	−0.139**	−0.152***	−0.141**	−0.154**
Total Scores	−0.491***	−0.458***	−0.445***	−0.496***

Results of the correlation between each dimensional variable of core self-evaluation and each dimensional variable of job burnout are shown in table 3. Results show significant negative correlations between three dimensions of job burnout and four dimensions of core self-evaluation (p<0.001).

**Table 3:** Correlation analysis between CSE and job burnout (r).

	Emotional Exhaustion	Depersonalization	Reduced Personal Accomplishment	Total Scores
Core self-evaluations	−0.334***	−0.322***	−0.371***	−0.369***

Each dimension of job burnout is used as the dependent variable in investigating the influence of the entire core self-evaluation on job burnout. Results show that core self-evaluation has a relatively positive predictive effect on emotional exhaustion, de-individuation, and lowered sense of personal achievement, see table 4.

**Table 4:** Regression analysis.

Independent	Dependent	*β*	*t*
Core self-evaluations	Emotional Exhaustion	−0.334	−7.465***
	Depersonalization	−0.322	−7.164***
	Reduced Personal Accomplishment	−0.322	−8.399***
	Total Score	−0.369	−8.363***

**Figure 1:**
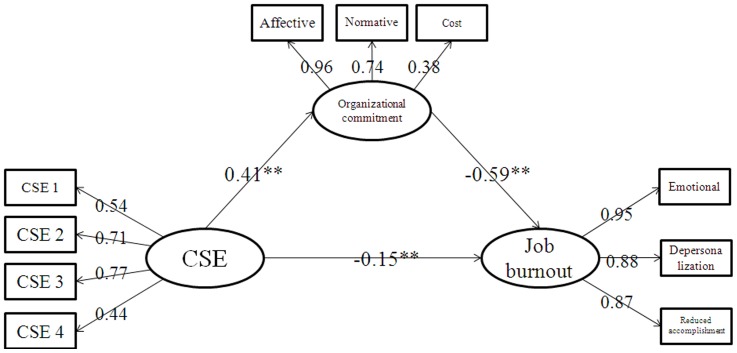
The whole model.

Based on above, we made a path analysis on the whole model, and the results were shown on figure 1. The following four indices were used to evaluate the goodness of fit of the model: (a) Chi-square statistic (χ^2^), χ^2^/df, (b) the Standardized Root Mean Square Residual (SRMR), (c) the Root Mean Square Error of Approximation (RMSEA), and (d) the Comparative Fit Index (CFI). In this study, a model was considered to have a good fit if all the path coefficients were significant at the level of 0.05, SRMR was below 0.08, RMSEA was below 0.08, and CFI was 0.95 or more. The results revealed the results showed that the model very good fit to the data, χ^2^ (26, N = 445) = 62.76, p<0.001; RMSEA = 0.048; SRMR = 0.069; and CFI = 0.0476.

At last we conducted the bootstrap test (a bootstrap sample of 1200 was specified), and the 95% confidence of the indirect effect from Core self-evaluations to job burnout through organizational commitment was from −0.162 to −0.319, which didn’t overlap with zero. The results together proved the mediating effect is significant.

## Discussion

### 4.1 Relationship between Core Self-evaluation and Organizational Commitment Of Nurses

A significant positive correlation exists between core self-evaluation and organizational commitment of nurses; i.e., the higher the nurses’ organizational commitment is, the higher their level of core self-evaluation. A possible reason for this finding is that the higher the employees’ core self-evaluation is, the higher their organizational identity and the larger their input and contribution. On the basis of this finding, managers should actively improve employees’ core self-evaluation and develop their potential. In turn, the organization will benefit from these efforts by obtaining higher revenue.

### 4.2 Relationship between Organizational Commitment and Job Burnout of Nurses

This research demonstrates that, although the correlation between lowered sense of personal achievement and continual commitment is not significant, a significant negative correlation exists between each dimension of organizational commitment and that of job burnout. This finding indicates that the higher the level of nurses’ organizational commitment is, the lower the level of their job burnout. Emotional commitment has a negative predictive effect on emotional exhaustion, de-individualization, and lowered sense of personal achievement, and continual commitment has a positive predictive effect on lowered sense of personal achievement. Therefore, the organizational commitment of nurses also has a considerable influence on job burnout. Other studies show a significant negative relationship between emotional exhaustion and organizational commitment. [9] Nursing is a typical vocation that deals with people and because of this, the longer nurses engage in this type of job, the higher their emotional exhaustion. Currently, nurses have a huge workload, but they are not properly compensated. Therefore, they harbor negative emotions that contribute to a perfunctory attitude toward work and that will negatively affect their organizational commitment over time. [12] This negativity severely cripples the working enthusiasm of the nursing staff, virtually setting a barrier between the nursing staff and the organization, and greatly affecting their contribution to the organization [17], [18]. In turn, their nursing quality decreases [19]. Therefore, research should take an active interest in nurses, and various ways and channels should be adopted to improve the level of nurses’ organizational commitment.

### 4.3 Relationship between Core Self-evaluation and Job Burnout of Nurses

Research findings show significant negative correlations in the three dimensions of job burnout and core self-evaluation. In other words, the higher the level of each dimension of nurses’ core self-evaluation is, the lower their sense of job burnout. Therefore, improving nurses’ core self-evaluation reduces job burnout, and lower job burnout improves self-esteem, locus of control, emotional stability, self-efficacy, and other aspects. Previous research reveals that increasing self-efficacy indicates a downtrend in the job burnout level [20], [21]. The self-confidence and enthusiasm toward work of nurses must be enhanced, and their ability to handle work-related stress must be improved. If nurses are optimistic and enthusiastic about work and have high endurance and adaptability, then their physical, emotional, and psychological exhaustion will be reduced, making them less likely to have job burnout.

### 4.4 Mediating Effect of Organizational Commitment on Nurses’ Core Self-evaluation and Job Burnout

This research constructs a complete model with core self-evaluation as the independent variable, job burnout as the dependent variable, and organizational commitment as the mediating variable. The model shows a good degree of fit. Based on the model, the load factor value on the path of core self-evaluation → job burnout is significant. This finding shows that core self-evaluation has a significant positive predicative effect on job burnout, and the direct effect between these two remains even after the intervention of organizational commitment. Therefore, both organizational commitment and core self-evaluation are able to influence job burnout, and organizational commitment plays a mediating role between them. Core self-evaluation is an individual-level feature, whereas organizational commitment is an organizational-level feature. When core self-evaluation influences job burnout through organizational commitment, a process that has to start from the individual and give back to the organization is completed to reduce job burnout.
